# Exploring barriers to seeking health care among Kenyan Somali women with female genital mutilation: a qualitative study

**DOI:** 10.1186/s12914-020-0222-6

**Published:** 2020-01-28

**Authors:** Samuel Kimani, Caroline W. Kabiru, Jacinta Muteshi, Jaldesa Guyo

**Affiliations:** 1 Africa Coordinating Centre for the Abandonment of FGM/C (ACCAF), University of Nairobi, P.O Box 19676-00202, Nairobi, Kenya; 2 School of Nursing Sciences, University of Nairobi, P.O Box 19676-00202, Nairobi, Kenya; 3 Population Council-Kenya, PO Box 17643-00500, Nairobi, Kenya

**Keywords:** Female genital mutilation or cutting, FGM/C, Barriers, Health care seeking, Somali, Kenya

## Abstract

**Background:**

Female genital mutilation/cutting (FGM/C) is a cultural practice associated with health consequences, women rights and deprivation of dignity. Despite FGM/C-related health consequences, circumcised women may encounter additional challenges while seeking interventions for reproductive health problems. Experiences of women/girls while accessing health services for reproductive health problems including FGM/C-related complications in poor, remote and hard to reach areas is poorly understood. We sought to explore barriers to care seeking among Somali women with complications related to FGM/C in public health facilities in Kenya.

**Methods:**

We drew on qualitative data collected from purposively selected women aged 15–49 years living with FGM/C, their partners, community leaders, and health providers in Nairobi and Garissa Counties. Data were collected using in-depth interviews (*n* = 10), key informant interviews (*n* = 23) and 20 focus group discussions. Data were transcribed and analyzed thematically using NVivo version 12.

**Results:**

Barriers were grouped into four thematic categories. Structural barriers to care-seeking, notably high cost of care, distance from health facilities, and lack of a referral system. Concerns regarding perceived quality of care also presented a barrier. Women questioned health professionals’ and health facilities’ capacity to offer culturally-sensitive FGM/C-specific care, plus ensuring confidentiality and privacy. Women faced socio-cultural barriers while seeking care particularly cultural taboos against discussing matters related to sexual health with male clinicians. Additionally, fear of legal sanctions given the anti-FGM/C law deterred women with FGM/C-related complications from seeking healthcare.

**Conclusion:**

Structural, socio-cultural, quality of service, and legal factors limit health seeking for reproductive health problems including FGM/C-related complications. Strengthening health system should consider integration of FGM/C-related interventions with existing maternal child health services for cost effectiveness, efficiency and quality care. The interventions should address health-related financial, physical and communication barriers, while ensuring culturally-sensitive and confidential care.

## Background

Defined as all procedures involving partial or total removal of the female external genitalia or other injuries for non-medical reasons, female genital mutilation/cutting (FGM/C) is a cultural practice associated with myriad health complications [1, 2]. The complications span from immediate, long-term physical, obstetric, gynecological, sexual and psychosocial impacts [2, 3]. The immediate complications including; severe pain, bleeding, and urine retention are related to the extent of the cutting, poor anatomical knowledge of the performer, use of crude and non-sterilized instruments [2–6]. The FGM/C-related gynaecologic complications entails menstrual retention, cysts, and infections (e.g genital, reproductive and urinary tract) [2, 5, 7–9]. Women with FGM/C also suffer obstetric complications such as difficult in birthing process, obstructed and prolonged labour, tears and episiotomies [4, 10, 11]. Of note are sexual consequences related to the severed organs notably painful sexual intercourse, lack of orgasm, satisfaction and lubrication [12–15]. Additionally, women living with FGM/C present with anxiety, depression, post-traumatic stress disorder, and low self-esteem - the so called psychological impacts [16–18]. Although FGM/C is associated with violation of bodily integrity, it is also constitute an extreme form of violence, abuse, and serious infringement on girls’ and women’s human rights [1, 19].

The World Health Organization (WHO) FGM/C typology documents four types of cutting [3]: type I - partial or total removal of the clitoris and/or the prepuce (glans and/or the body of the clitoris are cut) [20]; type II - partial or total removal of the clitoris and/or the prepuce (glans and/or the body of the clitoris are cut) as well as the labia minora, with or without excision of the labia majora (excision); type III - narrowing of the vaginal orifice with the creation of a covering seal by cutting and apposition or sewing together of the labia minora and/or the labia majora, with or without excision of the clitoris (infibulation) (type III); and type IV - all other harmful procedures to the female genitalia for non-medical reasons namely pricking, piercing, incising, scraping and cauterization [1]. The severity of FGM/C-related complications correlate with the extent of tissues cut or damaged and the type of FGM/C [21, 22].

Globally, an estimated 200 million women are living with FGM/C [23] with the heaviest burden borne by countries in Africa, the Middle East, and Asia. The practice is also reported in Latin America and some western nations that host immigrant communities from practicing countries [23, 24]. Nearly 3.6 million girls are at risk of being cut annually [23, 25]. In Kenya, approximately one out of every five women aged 15–49 years has undergone FGM/C [26]. However, the prevalence varies by region, ethnicity and other socio-demographic factors. While Kenyan FGM/C prevalence is at 21%, women (15–49 years) of Somali ethnicity have high rates of 94% of which a third (32%) of them have reportedly undergone type III FGM/C [26].

The Somalis are the largest Cushitic ethnic group in Kenya. Predominantly they are Muslims and resident of the former North Eastern Province of Kenya. They are a patriarchal society, with substantial power, authority and influence vested on men [27]. Historically, the Somali women practice type III (infibulation) FGM/C—also referred to as Pharaonic circumcision. Recently however, shift towards less severe cutting (Sunna circumcision) has been reported [28–31]. The community practices FGM/C to conform with culture and traditions, enhance girls’ marriageability, for aesthetics, avoid social sanctions, as well as for religious reasons [32–34]. The practice is alleged to ensure girl’s virginity and purity prior to marriage, an essential virtue for maintaining family’s honor and dignity [32, 35]. Indeed, infibulation is traditionally perceived as proof and a marker of virginity when young women get married [29, 30, 36]. However, unlike some ethnic groups in Kenya, FGM/C among the Somalis is not performed as a rite of passage to mark transition from childhood to adulthood [28].

Although women/girls living with FGM/C are at high risk for health complications [3, 23], they face additional challenges in care-seeking for the consequences and where available, it has been reported as sub-optimal [37–39]. Challenges in obtaining medical interventions for FGM/C-related complications are implicated with poor health statistics in communities with high prevalence [2, 4]. However, little is known on women’s experiences while accessing health care services for reproductive health problems including FGM/C-related complications in poor, remote and hard to reach areas where the practice is pervasive. This study sought to address this gap by examining the barriers to health-care seeking for FGM/C-related complications among Somali women living in both urban and rural settings in Kenya.

## Methods

### Study design

We drew on data from a larger cross-sectional qualitative study that sought to understand the shifts in FGM/C among families and health care providers from selected Kenyan communities that practice circumcision [29]. The study strived to understand why families sought FGM/C for their daughters from health care providers and why the providers accepted to cut the girls. The general context of FGM/C in the three Kenyan communities (Somali, Abagusii and Kuria) were elicited. Additionally, changes (shift) in FGM/C including cutting girls less severely and at younger age as well as medicalization were explored in the Somali and Abagusii communities residing in urban and rural settings, and among the Kuria in their rural setting. Data were collected between December 2016 and November 2018 using key informant interviews (KIIs), in-depth interviews (IDIs) and focus group discussions (FGDs) that had been developed for the wider aforementioned medicalization study. The discussion and interview guides contained broad questions that addressed the issues highlighted above, as well as probes that elicited specific FGM/C issues. For example, questions delved into specifics of immediate, obstetric, gynecologic, sexual and psychological complications. During the discussions/interviews, using the guides with general questions and probes the participants would outline the FGM/C-related consequences. The discussion/interview started with general statements followed with specific questions as the participant got comfortable. For example, in eliciting birth complications, a question would be pursued as follows, I want to hear about your own experience “what was your experience while delivering your first baby at the hospital”. Then, to capture the depth and breadth of FGM/C-related complications, probes would be used to elicit the specific details. “How did your experience of being cut affect your giving birth”. For example, from the aforementioned question, a probe would be, “you indicated having difficulties delivering your first baby because of cut … what did the doctors tell you … what did they do to assist you deliver”. Once the complications were identified, follow up questions on where the participants sought for medical help, and why there, as well as reasons that might have hindered them from seeking help were asked.

### Study location

For this paper, we used data collected from the Somali women, their partners, community leaders, and health care providers from Eastleigh (Nairobi County) and Garissa Township Constituency (Garissa County). Nairobi County hosts the capital city and has an ethnically diverse population with relatively high levels of education and modernization [29]. Neighborhoods such as Eastleigh are home to large population of Somalis who maintain very strong ties with their rural counterparts. On the other hand, Garissa Township Constituency comprises a mix of both urban and rural settings. Although the town is cosmopolitan, Somali is the dominant ethnic group. The target sites were purposively chosen, following stakeholder engagements on appropriateness of the location, research design as well as the likelihood of encountering Somali women living with type III FGM/C and related complications as well as sharing of experiences by the health care providers.

### Study participants

The study participants comprised women of reproductive age (15–49 years), mothers or female guardians with a daughter (s) who had experienced FGM/C-related complications, and health care providers (Additional file 1 and Additional file 2). Women were eligible for inclusion into the study if they were Somali, had undergone FGM/C, experienced FGM/C-related complications, and had sought care for FGM/C consequences from public health facilities as well as consented to the study. Husbands or male partners of women with FGM/C were also interviewed on the problems they associated FGM/C with on their partners/daughters as well as the role they played in helping them seek health care (Additional file 3). Additionally, community leaders were also interviewed (Additional file 4). As noted the prevalence of circumcision among the Kenyan Somali women is near universal [11], thus barriers encountered by women with FGM/C-related complications who sought medical help could be ascribed to FGM/C as well as some general reproductive health problems. However, for this study women were recruited based on their FGM/C status regardless of the circumciser. The FGM/C-related complications were defined as; those that occurred during the procedure such as pain and bleeding; birth complications associated with pin hole introitus including prolonged labour, tears and lacerations; gynecological complications namely difficult passing urine, keloid and scars; and sexual harms for example painful sexual intercourse, lack of satisfaction and orgasm. Furthermore, the health care providers (doctors, nurses, midwives, clinical officers, occupational therapist, trainee Nurse-Midwife and traditional birth attendants) working in public health facilities were interviewed to share their experiences while treating women with FGM/C-related complications (Additional file 5). Data from health care providers were triangulated with responses from community participants to generate a profile of barriers to health care seeking for FGM/C-related complications.

### Sampling and sample size

Study participants were recruited through purposive and snowball sampling based on the principles of qualitative research. The FGDs involved groups comprising of 6–12 participants. Separate FGDS were held for younger (younger than 30 years) and older (30 years and older) participants. The sample size was informed by thematic saturation of data during the discussions. A total of 20 FGDs, 23 KIIs and 10 IDIs were conducted with participants in the wider medicalization study involving the participants of Somali ethnicity. Thus, a subgroup of the participants from the wider medicalization study responded to the questions on the barriers to health seeking behavior among Somali women with FGM/C. There were ten healthcare providers interviewed including; occupational health (1), Clinical officers (3), Nurse-Midwives (5), trainee Nurse-Midwife (1), as well as five traditional birth attendants. A breakdown of the study participants is provided in Table 1.

**Table 1:** Overview of the study participants

Social demographic characteristics	Eastleigh			Garissa			Total
	FGDs	IDIs	KIIs	FGDs	IDIs	KIIs	
Community participants							
Male	4	1	9	4	2	3	23
Female	4	3	8	8	4	6	33
Total	8	4	17	12	6	9	56
Health-related professionals							
Nurse-Midwife			1			4	5
Clinical officers			3			0	3
Occupational Therapist			1			0	1
Trainee Nurse-Midwife			0			1	1
Traditional Birth Attendants			2			3	5
Community based leaders							
Religious leaders			2			1	3
Chief			1			2	3
Politician			0			1	1
Business man			2			0	2

### Recruitment, consent process and interview procedures

Locally networked community-based organizations and administrators were explained on the study objectives and the required participant characteristics to help recruit the initial participants from the community. Thereafter, snow-ball sampling through referrals from the initial participants was used to identify additional participants. As regards focus group discussions, the participants were identified through local organization contacts or administrators or hospital based recruitment after discussion about the characteristic required for the participants. Clients seeking health services were recruited by a health facility based research assistants with background and skills in Nursing. The health care providers were recruited from the health facilities by a health based research assistants with background and skills in Nursing or through referral from colleague health professionals. The recruitment was conducted after obtaining permission for the study from the director of health services as well as institutional head.

Once identified, permission granted, the participants were taken through the consenting process to understand: study components, risks involved, confidentiality, any compensation, and the freedom to withdraw from the interview without suffering or being punished. Thereafter, the participant was requested to sign or give a verbal consent to participate as well as allow for audio recording of the discussion/interview. Participants aged 18 years and older granted informed consent, while assent was obtained from younger (minors) participants, for which there was explanation on the study while the consent was provided by the parents or legal guardian for participation into the study and for audio recording. The discussions/interviews were conducted in private spaces and at all times convenient to the participants.

The interviews lasted for about 45 min while FGDs lasted one and a half hours. The discussions were conducted by a moderator and a recorder in a quiet and private space with no interruptions. During the discussions, the participants were seated in circle, with sessions commencing with introductions, after which an explanation on consent process and thereafter obtaining the consent from each FGD member. The moderator commenced the discussion by presenting questions on general FGM/C issues, then delving into specifics on FGM/C-related complications as well as health seeking challenges. The discussions were concluded by allowing contribution from every member until a consensus was reached. The discussions/interviews were conducted in Somali, Swahili or English depending on the language the participants were most comfortable with, while moderators/interviewers were trained locally-recruited research assistants. The interviews/discussions were audio recorded with some hand-written notes captured as well.

### Ethical considerations

Ethical approval for the study was granted by the Population Council’s Institutional Review Board (Ref: 775; dated: November 9, 2016) and the Kenyatta National Hospital-University of Nairobi (KNH-UoN; Ref: P527/07/2016; dated: October 12, 2016) and the National Commission for Science, Technology and Innovation (Ref: NACOSTI/P/16/79790/14328; dated: October 31, 2016). The permission to carry out the study in specific counties was granted by county administrators for Nairobi and Garissa counties and institutional heads, respectively.

### Data management and analysis

Demographic data were entered in anonymized form into password-protected Excel spreadsheets and descriptively analyzed. Digital audio recordings of the group discussions/ interviews were subjected to a multi-stage translation-transcription to ensure data quality. First, recordings in Somali and Kiswahili were transcribed verbatim by experienced Somali-or Kiswahili-speaking transcribers. Second, the anonymized transcripts were independently reviewed by target language-speaking translators, who checked the transcripts against the original audio recordings for accuracy, spellings and content. Any differences detected between the two formats were identified, discussed and resolved between the original transcription and the translator. Third, finalized transcripts were translated from Somali and Kiswahili to English by bilingual translators. A sample (10%) (*n* = 15) of the transcripts were reviewed by three independent reviewers and differences between the original English translation and the reviewed samples were identified and anomalies discussed until consensus on accurate translation was achieved. The finalized translated versions were then subjected to qualitative analyses. The transcripts were de-identified and stored in a password-protected computer. Transcripts and audio recordings were sent to the Population Council offices in Nairobi for archiving while audio recording were deleted.

The framework method for qualitative content analyses was adopted for this study [40]. The method is appropriate for thematic analysis of textual data where it is important to compare data by themes across many cases [41]. This approach combines deductive and inductive analyses of textual data with the flexibility to adapt emerging data and produce a coding framework, or ‘template’. The themes/codes were selected through a combined approach: deductively based on previous literature and the specifics of the research question from the interview guides, as well as inductively from the obtained data in the transcripts, followed by their refinement. From the literature, evidence show existence of barriers to health seeking among women with FGM/C-related complications that include: judgmental attitudes by health providers [32]; cultural incompetency [42, 43]; and poor communication [44, 45]. Clinicians were also noted to respond in shock, disbelief, as well as display of psychological and physical distress when they encountered women with FGM/C [32]. Related to aforementioned, stigmatization and ostracism on women with FGM/C by health care providers from non-cutting community affected health seeking [36, 38]. Additionally, the health care providers were reported to lack capacity to implement FGM/C-related management interventions as a result of poor or lack of training [46, 47]. The women with FGM/C also faced barriers to health seeking associated with lack of a well-functioning referral system [38, 43, 48].

The lead author and three research analysts developed a thematic coding framework through analysis of the study instruments and reading of the transcripts to reconcile and gain insights on emerging issues. The team reviewed the framework and definitions of each theme/code for consistency and understanding before commencement of coding using NVivo 12® [49]. Each analyst coded two transcripts to check the effectiveness and suitability of the coding framework. The team then refined the final coding framework. The analysts were then assigned the remaining transcripts. The emerging issues during coding were addressed during regular meetings. To ensure methodological rigor and trustworthiness of the study data, and in addition to the use of researcher triangulation—using three data analysts—and method triangulation—using three different interviews (IDIs, KIIs and FGDs)— inter-rater coding reliability was assessed by having analysts coding a random sample of two transcripts coded by another analyst. Differences in coding were compared, discussed and integrated by consensus.

## Results

**Fig. 1:**
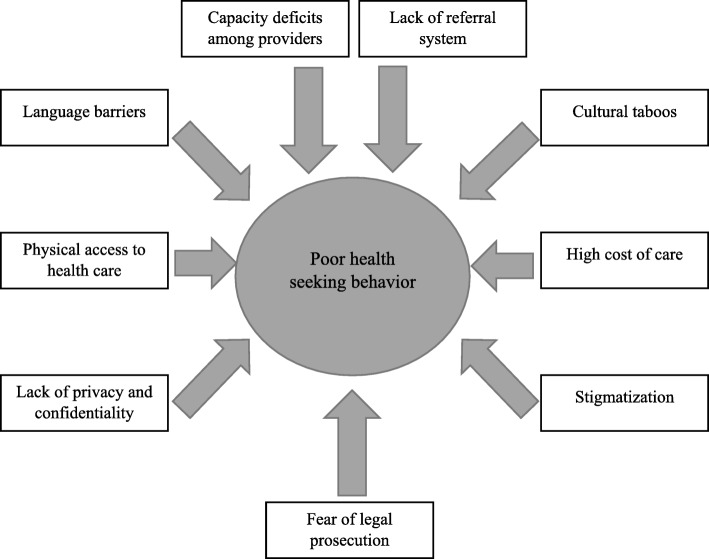
Summary of barriers to health seeking in public health facilities among Somali Women living with FGM/C in Kenya

Women living with FGM/C were reported to experience complications related to the cutting that required medical interventions. However, most of them were noted to present to the health facility when it was rather late, while some did not seek care at all resulting in high morbidity and mortality. Participants identified nine key barriers (sub-themes) to seeking health care for FGM/C-related complications. Emergent sub-themes from the data are summarized in Fig. 1. We ultimately grouped these sub-themes into four thematic categories: (1) Structural barriers to care-seeking, (2) concerns about the perceived quality of care, (3) socio-cultural barriers, and (4) the fear of legal sanctions.

### Structural barriers to care seeking

#### High cost of health services

Women and health care providers noted that the high cost of health services was a barrier to seeking healthcare for women with FGM/C-related complications. According to some participants, the high cost of health services contributed to women first seeking care from traditional healers, whose services were reportedly cheaper. Seeking care from a health facility was considered a last option decision, mainly when the traditional healers were unable to treat the condition or when the condition had worsened, as reported by a woman from Garissa:

“Women who have FGM-related complication seek first the assistance of traditional healers who are cheaper and available. They only go to the hospital as a last option when the condition does not seem to improve”. Married woman, FGD, Garissa

#### Limited physical access to facilities in difficult-to-reach areas

Physical access to health facilities (distance and location) can influence the uptake of health services. Women and girls who are living with FGM/C-related complications in rural areas were noted to face substantial challenges while accessing health care in public facilities compared to those in urban settings. Poor access to health facilities was suggested to result in delayed care seeking as quoted below:

“When ladies have health problems related to FGM especially in rural areas they seek help first from the herbalist because accessing health facilities is a big problem due long distance. The hospital will only be visited when it is too late when the problem has advanced” Married woman, IDI, Garissa

“If it is in town, the girls/women with complications go to hospitals and get treated. But if it’s in remote places she lives in ‘silac’ [extreme difficulties] and the complication might lead to death because accessing health facilities is such a big problem” Father to a cut girl, IDI, Eastleigh

#### Lack of referral system for FGM/C-related complications

Lack of a clear referral system for FGM/C-related complications was suggested to limit the uptake of health services. Health providers reported that women/girls with FGM/C-related complications faced challenges obtaining care because of the absence of a referral system. Most of patients were reported to be self-referral. The absence of clear referral pathway was also suggested to negatively affect the quality of care, with patients sent back and forth between facilities or being treated at facilities that lacked the capacity to manage the health complications. As a female nurse explained:

“Just recently someone was sent away from a City Council health facility and was told to go to a referral maternity hospital, she was moving up and down. Someone sent her back here [Health Centre]. We have a clinical officer who called me to assist her.” Female nurse, KII, Eastleigh

A project manager in Garissa also noted similar challenges:

There is no established system for referring women with complications associated with FGM, what we see is the women will be handled in the health facilities here regardless of whether they have the capacity to deal with the problems or not.” Project manager, KII, Garissa

### Concerns about the perceived quality of care

#### Stigmatization of women with FGM/C

Women suggested that FGM/C and the resultant complications exposed them to stigmatization from heath care providers working in public health facilities. The stigma arose when cut women and girls presented with “strange conditions” or disfigured external genitalia. Health care providers were noted to express shock and disgust. Some providers, particularly those in public facilities reportedly gossiped about the women or called the attention of their colleagues to come witness the “strange conditions,” which women considered a violation of their right to privacy. As one woman in Eastleigh explained:

“Our Somali community don’t go to government hospitals to give birth due to their conditions (FGM/C). They go to private facilities because in government facility, the health personnel will be shocked and even say this one has been cut”. Married woman, IDI, Eastleigh

#### Lack of privacy and confidentiality

Related to stigma, some of the women believed that interactions with health care providers were marred with the lack of privacy and confidentiality in the public health facilities. Breaches in confidentiality and privacy often arose when providers asked their colleagues to “come and see” the woman:

“In the government facility everyone wants to come see your private parts and then they express shock, get scared, and/or gossip about the external genitalia, However, in private it’s a matter of you, your money and your privacy”. Married Woman, FGD, Eastleigh

#### Capacity challenges among health care providers

The participants suggested that health care providers’ lacked skills to manage FGM/C-related health complications as a possible deterrent to health care seeking. This assertion was supported by a case narrative given by a health care provider who had encountered a client presenting with rare keloids related to FGM/C. As she explained, the client had sought help from various health facilities without success:

“I saw a woman who had stayed with keloids for 11 years, she passed on the other day in Kakuma [town in northwestern Turkana County]. She came all the way from Somalia to Dadaab [town in Garissa County, Kenya] and from Dadaab she went to Kakuma again. Her husband was telling us that every time doctors see her, they call other doctors come and see but nothing is done, and it kept on growing, it became very big” Female nurse, KII, Eastleigh

Some participants expressed frustrations from such behavior because neither the attending provider nor his or her colleagues would actually help the woman. As one woman reported:

“Women don’t seek medical help immediately and there are several reasons why they don’t go immediately. Like there is a woman who had a keloid who said she has been going to doctors, they only call their colleagues to come and see, but nothing was done to the problem”. Married Woman, IDI, Eastleigh

### Socio-cultural barriers

#### Language barriers

The Somali community is a closely-knit society and members mainly communicate in their native language [12]. Women reported that language barriers were a major obstacle to care seeking and patient-provider communication because of low literacy levels among some Somali women, particularly those from the rural areas. The absence of interpreters in the health facilities was noted to compound the problem. As a married woman reported:

“There is language barrier between the women and the health workers, especially being attended by a doctor who do not speak the Somali language.” Married Woman, IDI, Eastleigh

#### Cultural taboos

Some women living with FGM/C explained that the services received in health facilities were not culturally sensitive, which created a barrier to seeking care the next time they had a health need or when a relative had a problem that required medical intervention. For example, public health facilities were reportedly staffed with male health providers in the service delivery points that interfaced with women/girls living with FGM/C-related complications. According to the Somali culture, however, it is a taboo for a woman to discuss sexual health matters with another male except their husbands. Therefore, women found it difficult to discuss their medical concerns with male providers, particularly when they were from a different ethnic group. These challenges are common in public health facilities because of the government policy on diversification in recruitment, employment and deployment of public servants. A married woman in Nairobi explained:

“In the Somali community there are norms surrounding discussions on sexual matters and problems, for example being a woman and going to be attended by a male doctor and one who do not speak the Somali language is a taboo.” Married woman, IDI, Eastleigh

The nature and type of FGM/C-related complications also had a bearing on health seeking behavior because of the cultural taboo. Participants suggested that most Somali women and girls with FGM/C-related sexual complications failed to seek professional medical help in public health facilities. This was in contrast to women/girls who had physical or obstetric complications. As a traditional birth attendant explained:

“Like a girl with sexual problems related to FGM/C will keep the pain in secret, but the one with bleeding or unable to deliver the baby will be taken to the hospital without much delay” Traditional birth attendant, KII, Garissa

Similarly, a nurse in Nairobi stated:

“Somali women are shy to discuss sexual problems with a male doctor more so with those who are from other ethnicity. Your sex and sexuality in Somali community is a taboo you just do not talk anything to anybody anyhow”. Female nurse, KII, Eastleigh

### The fear of legal prosecution

The practice of FGM/C is illegal in Kenya under the Prohibition of Female Genital Mutilation Act of 2011 [29]. The practice however continues often under conditions of secrecy. Participants noted that the fear of facing legal sanctions deterred care seeking for FGM/C-related acute complications, particularly in public health facilities. Women and girls with FGM/C-related acute complications were therefore noted to present for care very late usually when the condition had deteriorated. Others were noted to completely avoid seeking health care. As an administrative officer in Nairobi noted:

“Girls/women with FGM-related complications only go to hospital because they don’t have any other alternative. Others hide because if they go to hospital they know they will be reported and jailed because FGM is prohibited by government.” Female Assistant Chief, KII, Eastleigh

A married woman in Garissa, similarly, stated:

“When women or girls have complications associated with FGM, they fear going to the health facilities but instead stay at home for fear of being reported to the police because FGM is illegal.” Married woman, FGD, Garissa

## Discussion

The findings provided an opportunity to better understand the challenges faced by Somali women/girls while seeking care for reproductive health problems including FGM/C-related complications in Kenyan public health facilities. Four main categories of barriers were identified: structural, perceived quality of care, socio-cultural, and fear of legal prosecution. Although the findings relate to women with FGM/C-related complications, they also demonstrate how the state of health care can impede care seeking in women with reproductive health problems. Women/girls with FGM/C may face additional unique challenges and needs calling for strengthening of health system through integration of FGM/C interventions into existing reproductive services.

At the structural level, the cost of care was noted to be prohibitively high. Health services are expensive in Kenya [43] in terms of direct and indirect costs. Lack of integrated model for health services characterized by perennial shortages, long distance from facility, waiting time as well as challenges in financing are attributed to expensive care. Additionally, inability to purchase health insurance, limited clients’ ability to cover out-of-pocket health expenses associated with poverty affect access to care. To forestall these challenges, Kenya is currently piloting a universal health care model in line with the national development agenda set out by the President of Kenya—the Big Four agenda—to improve access and financing of care [43, 44]. The model is envisaged to promote human rights for women/girls, as well as accelerate achievement of the Sustainable Development Goals (SDGs). Recently, findings from Somaliland showed utilization of health services for FGM/C-related complications [45] was hindered by high cost. High cost may deter women/girls with FGM/C from seeking immediate care or turn to non-medical practitioners jeopardizing their health. This would negated the WHO best principles for enhancing care for cut women and girls [3]. As Kenya expands its universal health care model, increasing access for cut women/girls by considering cost barriers should be a priority intervention. The considerations should also address challenges with long distances and poor transport system especially in the rural, and hard to reach areas like Garissa county [43]. Interventions to improve access to health services, such as outreach/mobile clinics, can be promising investments in these areas where FGM/C is pervasive. The national and county governments should leverage on existing programs such as the Beyond Zero Campaign mobile clinics [46] to integrate FGM/C- related management and prevention interventions.

Kenyan health system experiences challenges in responding to the burden of FGM/C as evidenced by poor or lack of documentation, absence of an audit system on FGM/C cases, complications, and referrals [47]. Not surprisingly therefore, lack of an effective referral system for further care of FGM/C cases was highlighted as a challenge. Indeed, inadequate or absence of referral system affect access to care for women requiring reproductive health services [48, 50]. Thus, strengthening the health system to ensure availability and accessibility of FGM/C care services including referrals for specialized interventions is critical in mitigating the impacts of FGM/C.

Stigmatization with its elements, notably perceived discrimination/stereotyping and culture shock [51], has been flagged as a deterrent to seeking medical care in women with FGM/C-related complications [52]. We found that, women seeking care for FGM/C-related complications, faced stigma while seeking medical services. Consistent with other studies [32] health care providers were noted to express shock, disgust, and disbelief upon encountering women with FGM/C. Similar reaction characterized with stigmatization and ostracism towards women with FGM/C has been reported among professionals from non-cutting communities or communities that perform a different type of FGM/C [36, 38]. Such reactions disregard the feelings of women who have perceived their genitalia to be normal since childhood – the so called normalization [32]. The phenomenon has two elements - the cultural normalization of FGM/C, as well as living with symptoms of FGM/C-related complications for months or even years without seeking care despite the associated pain and health risks [6, 53]. Normalization of FGM/C-related complications has been linked to barriers of access to specialized care, shame of seeking services, not associating the complications to FGM/C as well as knowledge of other female community members with similar conditions [6]. Although cultural normalization is prevalent, it does not minimize FGM/C-related complications such as the mental health consequences [54] [3] calling for tactful inquiry regarding long term consequences by the health care providers.

Related to the aforementioned, reactions towards complications may stem from providers’ limited knowledge of FGM/C. Concerns about stigma and providers’ limited capacity to handle FGM/C-related complications underscore the need to strengthen the health system response to FGM/C [6, 55, 56]. Importantly, the interventions must be holistic, sensitive, multidisciplinary [57], and should include education on the importance of avoiding verbal and non-verbal reactions that may trigger feeling of stigmatization [58] in institutions that ought to be in the frontline promoting dignity and human rights. From the aforementioned, we recommend the inclusion of FGM/C modules in the trainee health care providers curricula and development of innovative trainings/professional development for those working to be implemented to address most of the barriers highlighted in this paper.

Strengthening the training of health care providers is a critical intervention because capacity deficits limit the uptake of health services [59, 60]. Importantly, there are numerous support tools [46] for management of FGM/C-related complications as well as prevention of new FGM/C cases [2, 47, 61, 62]. These tools can be useful in addressing gaps in knowledge and skills among health care providers. Although the tools are developed by the World Health Organizations, countries ministries of health in high prevalent FGM/C practices should adopt, adapt and customize the tools to their settings.

Communication barriers between clients and providers are associated with suboptimal care [51, 63, 64]. Impaired communication associated with language differences was highlighted as a concern for the clients with low literacy levels. Most of health care providers were not from the local communities and could not understand or speak in local languages. Similar communication challenges that hindered access to care were reported among Somali women seeking asylum in Europe [59, 65, 66]. To address the challenge, family members would act as interpreters. However, the aforementioned may compromise patient confidentiality calling for the use of ‘cultural brokers’ or interpreters [67]. While the recommendation may suffice for Western countries, its applicability in Kenya need evaluation. The current constitutional dispensation has health as a devolved function with the mandate of recruitment and deployment. Thus the devolved governance and the universal health care model, gives an opportunity to mainstream community health workers and volunteers into the health system that could address many challenges including communication.

Cultural incompetency and insensitivity among health professionals as barriers to health seeking have been documented extensively [66, 68, 69]. Our study showed that health service provision was not sensitive to patients’ cultural background. For instance, service delivery points that interfaced with women/girls with FGM/C-related sexual complications were reportedly staffed by male providers; yet, among the Somalis it is a taboo to discuss issues around sexuality/sexual experiences with another man other than one’s husband. Cultural incompetence and insensitivity is a major barrier to seeking obstetrics, gynecological, urinary and reproductive-related services [70]. Indeed, it compromises confidence in response and prevention of FGM/C [71–73]. Thus, a clarion call for implementers of interventions for FGM/C-related management and prevention, is to familiarize themselves with culture and customs [74–76] for competent response [77] and continuously adjust to offer appropriate and respectful care [78].

Women with acute FGM/C-related complications faced additional fear that presenting for care exposed them to legal sanctions because the practice is illegal in Kenya [42]. This is consistent with evidence that fear of criminalization limits access to much needed health services [79, 80]. Healthcare providers have a responsibility to take the legislative framework into consideration and may be required to report FGM/C cases; thus, facing an ethical dilemma. As outlined in WHO guidelines for the management of FGM/C-related health complications, “states must set in place systems and structures to support ‘women and children who are victims of harmful practices’ by ensuring access to ‘immediate support services, including medical, psychological and legal services’” (page 7) [47].

### Limitations

Study findings should be interpreted in light of some limitations. First, the prevalence of FGM/C among the Kenyan Somali women is near universal, as much as we attribute the reported barriers of health seeking to FGM/C-related complications, the same can be applicable to the general reproductive health problems. However, these are the first findings to shed light on barriers faced by women with FGM/C-related complications in marginalized as well as the urban settings, highlighting a special gap that need to be addressed. In addressing the health seeking barriers, integration of health care services would be a promising approach to bridge the gap. Second, although it was easy to ask and get feedback on acute FGM/C-related complications because of recall, there may be difficulties for long-term ones because clients may have normalized them because FGM/C is a social norm and women may be living with related complications without seeking care. However, our interview/discussion approach of commencing with general statements then specific probes helped to elicit the information regarding the complications. Third, the study draws on data from only one community and two study sites thus, the findings may not be generalizable. Despite the aforementioned, our study findings shed important light on key factors that might limit health service utilization for women and girls living with FGM/C and by extension other reproductive health challenges in both urban and rural settings. This calls for integrated health services that address the high risk of morbidity and mortality that may be associated to barriers to health seeking for women and girls.

## Conclusion

Women and girls living with FGM/C face numerous challenges while seeking medical interventions for reproductive health problems in Kenyan public health facilities. The challenges are broadly categorized into structural, quality of care, socio-cultural, and legal barriers. These barriers limit the uptake of potentially lifesaving care for FGM/C-related complications and expose women/girls to the risk of long-term morbidity. Study findings underscore the need to strengthen the health system through integration of interventions for FGM/C-related prevention and management of women/girls living with FGM/C. In particular, there is need for interventions that facilitate access to holistic affordable health services; that strengthen health care providers’ capacity to offer competent, culturally-sensitive and confidential care; and that protect health providers from legal sanctions for providing critical health services to those living with FGM/C. A multi-disciplinary approach should incorporate health, social, and legal practitioners to ensure policies and services respond to the reproductive health needs and support the well-being of women/girls. A need to improve evidence generation to guarantee availability of data that should inform targeted investments, service provision, and health response to the needs of women and girls is critical.

## Supplementary information

**Additional file 1.** Focus group discussion guide.

**Additional file 2.** In depth interview guide for mothers, female guardians (Medicalization/shifts in FGM/C).

**Additional file 3.** In depth interview guide for Husbands, Male partners (Medicalization/shifts in FGM/C).

**Additional file 4.** Key informant interview guide for community leaders (Medicalization/shifts in FGM/C).

**Additional file 5.** Key informant interview guide for Health care providers (Medicalization/shifts in FGM/C).

## Data Availability

The datasets analysed during the current study are not yet publicly available but are available from the corresponding author on reasonable request.

## Abbreviations

FGD: Focus group discussion
FGM/C: Female genital mutilation or cutting
IDI: In-depth interview
KII: Key informant interview
SDG: Sustainable Development Goal

## References

[1] WHO. Eliminating female genital mutilation: An interagency statement. 2008. http://www.unfpa.org/sites/default/files/pub-pdf/eliminating_fgm.pdf. Accessed 4 Jan 2019.

[2] Kimani S, Muteshi J. Health Impacts of Female Genital Mutilation/Cutting: A Synthesis of the Evidence. 2016:[29 p.]. https://www.popcouncil.org/uploads/pdfs/2016RH_HealthImpactsFGMC.pdf. Accessed 3 Sept 2019.

[3] WHO. WHO guidelines on the management of health complications from female genital mutilation 2016. Available from: http://apps.who.int/iris/bitstream/10665/206437/1/9789241549646_eng.pdf?ua=1. Accessed 3 Sept 2019. 27359024

[4] WHO (2006). Female genital mutilation and obstetric outcome: WHO collaborative prospective study in six African countries. Lancet.

[5] Berg Rigmor C, Underland Vigdis, Odgaard-Jensen Jan, Fretheim Atle, Vist Gunn E (2014). Effects of female genital cutting on physical health outcomes: a systematic review and meta-analysis. BMJ Open.

[6] WHO. Care of girls and women living with female genital mutilation: a clinical handbook. 2018:[458 p.]. https://apps.who.int/iris/bitstream/handle/10665/272429/9789241513913-eng.pdf. Accessed 3 Sept 2019.

[7] Collinet P, Sabban F, Lucot J, Boukerrou M, Stien L, Leroy J (2004). Management of type III female genital mutilation. J Gynecol Obstet Biol Reprod (Paris).

[8] Nour Nawal (2015). Female Genital Cutting: Impact on Women's Health. Seminars in Reproductive Medicine.

[9] Utz-Billing I, Kentenich H (2008). Female genital mutilation: an injury, physical and mental harm. J Psychosom Obstet Gynecol.

[10] Berg RC, Underland V (2013). The obstetric consequences of female genital mutilation/cutting: a systematic review and meta-analysis. Obstet Gynecol Int.

[11] Varol N, Dawson A, Turkmani S, Hall JJ, Nanayakkara S, Jenkins G (2016). Obstetric outcomes for women with female genital mutilation at an Australian hospital, 2006–2012: a descriptive study. BMC Pregnancy Childbirth.

[12] Connor JJ, Hunt S, Finsaas M, Ciesinski A, Ahmed A, Robinson BBE (2016). Sexual health care, sexual behaviors and functioning, and female genital cutting: perspectives from Somali women living in the United States. J Sex Res.

[13] Abdulcadir J, Botsikas D, Bolmont M, Bilancioni A, Djema DA, Bianchi Demicheli F (2016). Sexual anatomy and function in women with and without genital mutilation: a cross-sectional study. J Sex Med.

[14] Esho T, Kimani S, Nyamongo I, Kimani V, Muniu S, Kigondu C (2017). The ‘heat’ goes away: sexual disorders of married women with female genital mutilation/cutting in Kenya. Reprod Health.

[15] Abdel-Aleem MA, Elkady MM, Hilmy YA (2016). The relationship between female genital cutting and sexual problems experienced in the first two months of marriage. Int J Gynaecol Obstet.

[16] Mulongo P, Hollins Martin C, McAndrew S (2014). The psychological impact of female genital mutilation/cutting (FGM/C) on girls/women’s mental health: a narrative literature review. J Rep Infant Psychol.

[17] Ahmed MR, Shaaban MM, Meky HK, Amin Arafa ME, Mohamed TY, Gharib WF (2017). Psychological impact of female genital mutilation among adolescent Egyptian girls: a cross-sectional study. Eur J Contracept Reprod Health Care.

[18] Berg RC, Denison E, Fretheim A (2010). Psychological, social and sexual consequences of female genital mutilation/cutting (FGM/C): a systematic review of quantitiative studies.

[19] Reza A, Mercy JA, Krug E (2001). Epidemiology of violent deaths in the world. Inj Prev.

[20] Abdulcadir J, Margairaz C, Boulvain M, Irion O (2011). Care of women with female genital mutilation/cutting. Swiss Med Wkly.

[21] Agugua N, Egwuatu V (1982). Female circumcision: management of urinary complications. J Trop Pediatr.

[22] Aziz F (1980). Gynecologic and obstetric complications of female circumcision. Int J Gynecol Obstet.

[23] UNICEF. Female Genital Mutilation/Cutting: A Global Concern. 2016. https://www.unicef.org/media/files/FGMC_2016_brochure_final_UNICEF_SPREAD.pdf. Accessed 20 Sept 2019.

[24] Yoder PS, Wang S, Johansen E (2013). Estimates of female genital mutilation/cutting in 27 African countries and Yemen. Stud Fam Plan.

[25] UNFPA, UNICEF. Joint Evaluation of the UNFPA-UNICEF Joint Programme on Female Genital Mutilation/Cutting: Accelerating Change Country Case Studies. 2013. https://www.unfpa.org/sites/default/files/admin-resource/FGM-report%2012_4_2013.pdf. Accessed 3 Sept 2019.

[26] Kenya National Bureau of Statistics, ICF Macro (2014). Kenya Demographic and Health Survey 2014.

[27] El-Bushra J. Gender and Forced Migration: Editorial. Forced Migration Review, 9th Edition [Internet]. 2000. https://www.fmreview.org/sites/fmr/files/FMRdownloads/en/gender-and-displacement/elbushra.pdf. Accessed 4 Jan 2019.

[28] Jaldesa GW, Askew I, Njue C, Wanjiru M. Female genital cutting among the Somali of Kenya and management of its complications; 2005. http://www.popcouncil.org/pdfs/frontiers/FR_FinalReports/Kenya_Somali.pdf.

[29] Kimani S, Kabiru C. Shifts in female genital mutilation/cutting: perspectives of families and health care providers. Evidence to end FGM/C: research to help women thrive [internet]. 2018. http://www.popcouncil.org/EvidencetoEndFGM-C. Accessed 3 Sept 2019.

[30] Powell R, Yussuf M (2018). Changes in FGM/C in Somaliland: medical narrative driving shifts in types of cutting.

[31] Shell-Duncan B, Njue C, Moore Z. The Medicalization of Female Genital Mutilation /Cutting: What do the Data Reveal? Evidence to End FGM/C: Research to Help Women Thrive [Internet]. 2017. https://www.popcouncil.org/uploads/pdfs/2017RH_MedicalizationFGMC.pdf. Accessed 4 Jan 2018.

[32] Horowitz CR, Jackson JC (1997). Female "circumcision": African women confront American medicine. J Gen Intern Med.

[33] Rouzi AA (2013). Facts and controversies on female genital mutilation and Islam. Eur J Contracept Reprod Health Care.

[34] Wahlberg A, Johnsdotter S, Ekholm Selling K, Källestål C, Essén B (2017). Factors associated with the support of pricking (female genital cutting type IV) among Somali immigrants – a cross-sectional study in Sweden. Reprod Health.

[35] Mackie G, LeJeune J (2009). Social dynamics of abandonment of harmful practices: a new look at the theory: UNICEF.

[36] Johnsdotter S, Essén B (2016). Cultural change after migration: circumcision of girls in Western migrant communities. Best Pract Res Clin Obstet Gynaecol.

[37] Evans C, Tweheyo R, McGarry J, Eldridge J, McCormick C, Nkoyo V (2017). What are the experiences of seeking, receiving and providing FGM-related healthcare? Perspectives of health professionals and women/girls who have undergone FGM: protocol for a systematic review of qualitative evidence. BMJ Open.

[38] Mbanya VN, Gele AA, Diaz E, Kumar B (2018). Health care-seeking patterns for female genital mutilation/cutting among young Somalis in Norway. BMC Public Health.

[39] Bjalkander O, Bangura L, Leigh B, Berggren V, Bergstrom S, Almroth L (2012). Health complications of female genital mutilation in Sierra Leone. Int J Women's Health.

[40] Shell-Duncan B (2001). The medicalization of female “circumcision”: harm reduction or promotion of a dangerous practice?. Soc Sci Med.

[41] Ritchie J, Lewis J, Nicholls CMN, Ormston R (2013). Qualitative research practice: a guide for social science students and researchers: SAGE publications.

[42] Dawson A, Turkmani S, Fray S, Nanayakkara S, Varol N, Homer C (2015). Evidence to inform education, training and supportive work environments for midwives involved in the care of women with female genital mutilation: a review of global experience. Midwifery.

[43] Varol N, Hall JJ, Black K, Turkmani S, Dawson AJRH (2017). Evidence-based policy responses to strengthen health, community and legislative systems that care for women in Australia with female genital mutilation / cutting.

[44] Berggren Vanja, Abdel Salam Gerais, Bergström Staffan, Johansson Eva, Edberg Anna-Karin (2004). An explorative study of Sudanese midwives’ motives, perceptions and experiences of re-infibulation after birth. Midwifery.

[45] Lazar Jalana N., Johnson-Agbakwu Crista E., Davis Olga I., Shipp Michele P.-L. (2013). Providers' Perceptions of Challenges in Obstetrical Care for Somali Women. Obstetrics and Gynecology International.

[46] Abdulcadir J, Say L, Pallitto C (2017). What do we know about assessing healthcare students and professionals' knowledge, attitude and practice regarding female genital mutilation? A systematic review. Reprod Health.

[47] Kimani S, Esho T, Kimani V, Muniu S, Kamau J, Kigondu C (2018). Female genital mutilation/cutting: innovative training approach for nurse-midwives in high prevalent settings. Obstet Gynecol Int.

[48] Moxey JM, Jones LL (2016). A qualitative study exploring how Somali women exposed to female genital mutilation experience and perceive antenatal and intrapartum care in England. BMJ Open.

[49] Gale NK, Heath G, Cameron E, Rashid S, Redwood S (2013). Using the framework method for the analysis of qualitative data in multi-disciplinary health research. BMC Med Res Methodol.

[50] Bazeley P (2007). Qualitative data analysis with NVivo: SAGE publications.

[51] National Council for Law Reporting (2011). Prohibition of female genital mutilation act. No. 32 of 2011.

[52] Mugo P, Onsomu E, Munga B, Nafula N, Mbithi J, Owino E (2018). Phares Mugo, Eldah Onsomu, Boaz Munga, Nancy. Nafula, Juliana Mbithi and Esther Owino.

[53] Mackie G (2000). Female genital cutting: the beginning of the end. Female “circumcision” in africa: culture, controversy, and change.

[54] Mulongo P, McAndrew S, Hollins MC (2014). Crossing borders: discussing the evidence relating to the mental health needs of women exposed to female genital mutilation. Int J Ment Health Nurs.

[55] Isman E, Mahmoud Warsame A, Johansson A, Fried S, Berggren V. Midwives’ experiences in providing care and counselling to women with female genital mutilation (FGM) related problems. Obstet Gynecol Int. 2013;2013. 10.1155/2013/785148 PMC3791569 24163698

[56] BeyondZero. Beyond Zero. 2013. https://www.beyondzero.or.ke/. Accessed 4 Sept 2019.

[57] Mujasi PN, Asbu EZ, Puig-Junoy J (2016). How efficient are referral hospitals in Uganda? A data envelopment analysis and tobit regression approach. BMC Health Serv Res.

[58] Higginbottom G, Safipour J, Yohani S, O'Brien B, Mumtaz Z, Paton P (2016). An ethnographic investigation of the maternity healthcare experience of immigrants in rural and urban Alberta, Canada. BMC Pregnancy Childbirth.

[59] Fried S, Mahmoud Warsame A, Berggren V, Isman E, Johansson A (2013). Outpatients’ perspectives on problems and needs related to female genital mutilation/cutting: a qualitative study from Somaliland. Obstet Gynecol Int.

[60] Balfour J, Abdulcadir J, Say L, Hindin MJ (2016). Interventions for healthcare providers to improve treatment and prevention of female genital mutilation: a systematic review. BMC Health Serv Res.

[61] Johnson-Agbakwu C, Warren N (2017). Interventions to address sexual function in women affected by female genital cutting: a scoping review. Curr Sex Health Rep.

[62] Johnsdotter S (2018). The impact of migration on attitudes to female genital cutting and experiences of sexual dysfunction among migrant women with FGC. Curr Sex Health Rep.

[63] Perron Liette, Senikas Vyta, Burnett Margaret, Davis Victoria, Burnett Margaret, Aggarwal Anjali, Bernardin Jeanne, Clark Virginia, Davis Victoria, Fisher William, Pellizzari Rosana, Polomeno Viola, Rutherford Maegan, Sabourin Jeanelle, Shapiro Jodi, Akhtar Saima, Camire Bruno, Christilaw Jan, Corey Julie, Nelson Erin, Pierce Marianne, Robertson Deborah, Simmonds Anne (2013). Female Genital Cutting. Journal of Obstetrics and Gynaecology Canada.

[64] Leye Els, Ysebaert Ilse, Deblonde Jessika, Claeys Patricia, Vermeulen Gert, Jacquemyn Yves, Temmerman Marleen (2008). Female genital mutilation: Knowledge, attitudes and practices of Flemish gynaecologists. The European Journal of Contraception & Reproductive Health Care.

[65] Zenner N, Liao L-M, Richens Y, Creighton S (2013). Quality of obstetric and midwifery care for pregnant women who have undergone female genital mutilation. J Obstet Gynaecol.

[66] Doucet M-H, Pallitto C, Groleau D (2017). Understanding the motivations of health-care providers in performing female genital mutilation: an integrative review of the literature. Reprod Health.

[67] Rouzi AA, Alturki F (2015). Female genital mutilation/cutting: an update. Clin Exp Obstet Gynecol.

[68] Crooks VA, Hynie M, Killian K, Giesbrecht M, Castleden H (2011). Female newcomers’ adjustment to life in Toronto, Canada: sources of mental stress and their implications for delivering primary mental health care. GeoJournal.

[69] Widmark C, Leval A, Tishelman C, Ahlberg BM (2010). Obstetric care at the intersection of science and culture: Swedish doctors' perspectives on obstetric care of women who have undergone female genital cutting. J Obstet Gynaecol.

[70] Higginbottom G, Safipour J, Mumtaz Z, Chiu Y, Paton P, Pillay JJBP, et al. “I have to do what I believe”: Sudanese women’s beliefs and resistance to hegemonic practices at home and during experiences of maternity care in Canada. BMC Pregnancy Childbirth. 2013;13(1):51. 10.1186/1471-2393-13-51 PMC3599128 23442448

[71] Jacoby SD, Lucarelli M, Musse F, Krishnamurthy A, Salyers V (2015). A mixed-methods study of immigrant Somali Women's health literacy and perinatal experiences in Maine. J Midwifery Womens Health.

[72] Kingston D, Heaman M, Chalmers B, Kaczorowski J, O'Brien B, Lee L (2011). Comparison of maternity experiences of Canadian-born and recent and non-recent immigrant women: findings from the Canadian maternity experiences survey. J Obstet Gynaecol Can.

[73] Reitmanova S, Gustafson DL (2008). "they can't understand it": maternity health and care needs of immigrant Muslim women in St. John's, Newfoundland. Matern Child Health J.

[74] Allotey P, Manderson L, Grover S (2001). The politics of female genital surgery in displaced communities. Crit Public Health.

[75] Dawson A, Homer CS, Turkmani S, Black K, Varol N (2015). A systematic review of doctors' experiences and needs to support the care of women with female genital mutilation. Int J Gynaecol Obstet.

[76] Dawson A, Turkmani S, Varol N, Nanayakkara S, Sullivan E, Homer C (2015). Midwives’ experiences of caring for women with female genital mutilation: insights and ways forward for practice in Australia. Women Birth.

[77] Babalola S, Brasington A, Agbasimalo A, Helland A, Nwanguma E, Onah N (2006). Impact of a communication programme on female genital cutting in eastern Nigeria. Tropical Med Int Health.

[78] Diop NJ, Askew I (2009). The effectiveness of a community-based education program on abandoning female genital mutilation/cutting in Senegal. Stud Fam Plan.

[79] Richard F, Ahmed W, Denholm N, Dawson A, Varol N, Essén B (2017). Female genital mutilation/cutting: sharing data and experiences to accelerate eradication and improve care: part 2. Reprod Health.

[80] Johnson RL, Saha S, Arbelaez JJ, Beach MC, Cooper LA (2004). Racial and ethnic differences in patient perceptions of bias and cultural competence in health care. J Gen Intern Med.

