# Supplementary material for: Exploring barriers to seeking health care among Kenyan Somali women with female genital mutilation: a qualitative study
Source: BMC Int Health Hum Rights. 2020 Jan 28;20:3. doi: 10.1186/s12914-020-0222-6 (PMC6986153; doi:10.1186/s12914-020-0222-6)
Supplement: Supplementary file 1 — Additional file 1. Focus group discussion guide. [file 12914_2020_222_MOESM1_ESM.doc]

Additional File 1: FOCUS GROUP DISCUSSION GUIDE

Understanding Medicalization of Cutting through Qualitative Research on Families Using Medicalized FGM/C and Medical Professionals Providing Medicalized Services

Focus Group Guidelines (designed to illuminate FGM/C-related complications and barriers to seeking care)

Focus group discussion guide for families that have experience with FGM/C. Please use this guide to facilitate the focus group discussion with members of community that have experienced FGM/C. The FGD will elicit information concerning knowledge, complications and barrier to seeking services for the complications. The information collected should be backed up by the participants’ experience/evidence.

[Note: Separate focus groups can be held with 1) young mothers, 2) older women, 3) young girls who are just past age of female circumcision]

Guidelines for Focus Group Discussions

| Topic 1. General knowledge on FGM/C | | |
| --- | --- | --- |
|  | 1.1 | What is your opinion about FGM in this community?  PROBE: Type of FGM; How common is FGM; Age of cutting; Decreasing or Not; Is it still important; Who is supporting it and who is not |
| Topic 2. Complications associated with FGM/C | | |
|  | 2.1 | What complications have you experienced or come across or heard associated with FGM  PROBE FOR: Immediate complications; Gynaecological complications; Urological complications; Obstetric complications; Sexual complications; Psychological complication; Social complications |
| Topic 3. Health seeking behaviour of women with complications | | |
|  | 3.1 | Where do women or girls with FGM-related complications go to seek help for their problems?  PROBE: Traditional healers; Public health facilities; Private health facilities; Call health personnel to the girls/woman house, Stay at home |
|  | 3.2 | What are the barriers to seeking medical help from public health facilities by women or girls with FGM related complications?  Probe for: cost, distance, timeliness, attitude of health care providers, others |
|  | 3.3 | What do you suggest should be done to health facilities so that more women with FGM/C complications can seek for help?  PROBE FOR: |
|  |  | Is there anything else you would want to add??? |
