# Supplementary material for: Exploring barriers to seeking health care among Kenyan Somali women with female genital mutilation: a qualitative study
Source: BMC Int Health Hum Rights. 2020 Jan 28;20:3. doi: 10.1186/s12914-020-0222-6 (PMC6986153; doi:10.1186/s12914-020-0222-6)
Supplement: Supplementary file 2 — Additional file 2. In depth interview guide for mothers, female guardians (Medicalization/shifts in FGM/C). [file 12914_2020_222_MOESM2_ESM.doc]

# **Additional File 2: In depth interview guide for mothers, female guardians (Medicalization/shifts in FGM/C)**

In depth interview (designed to illuminate FGM/C-related complications and barriers to seeking care)

In depth interview (IDI) guide for families that have experience with FGM/C. Please use this guide to facilitate the IDI with mothers/female guardians that have experienced FGM/C. The IDI will elicit information concerning knowledge, complications and barrier to seeking services for the complications. The information collected should be backed up by the participants’ experience/evidence.

**Individual mothers, female guardians**

In-depth interview number:

In-depth Interview date:

Narrative Interviewer’s name:

Ethnicity of the participant:

Gender of the participant:

Age of participant:

Marital status of participant:

Number of children (boys/girls) of the participant:

Number of years lived in community:

Other relevant demographic information of participant:

Language in which narrative interview was undertaken:

Informed Consent given by participant (Purpose of the interview and confidentiality explained. Informed Consent forms signed and collected:

Consent given by participant to audio record the narrative interview:

Participants’ copy of informed consent form given:

***Introduction:***

1. Please tell me a little bit about yourself and your role/position/status in your family?

***Knowledge of FGM/C:***

*[NOTE: This section should provide information concerning the participant’s knowledge of FGM/C.]*

1. What would you say about FGM/C in your community? (Explore issues as appropriate without placing participant in a situation where they feel uncomfortable).
2. What is the principal type of FGM /C performed in your community?
3. Why do girls have to go through FGM/C
4. Where do people go to have FGM/C done?
5. What is the justification for FGM/C in your community? [**PROBE FOR**: If marriage, religion, and social pressure are mentioned, probe what beliefs underpin each of these and whether they think they are still relevant today?]
6. What complications have you experienced or come across or heard associated with FGM

**PROBE FOR:** Immediate complications; Gynaecological complications; Urological complications; Obstetric complications; Sexual complications; Psychological complication; Social complications

1. Where did/do you go or women or girls with FGM-related complications go to seek help for their problems? PROBE: Traditional healers; Public health facilities; Private health facilities; Call health personnel to the girls/woman house, Stay at home
2. What barriers did you encounter while seeking medical help from public health facilities because of FGM related complications? Probe for: cost, distance, timeliness, attitude of health care providers, others
3. What do you suggest should be done to health facilities so that more women with FGM/C complications can seek for help? PROBE FOR:
4. Do you have any comments you would like to add?
