# Supplementary material for: Exploring barriers to seeking health care among Kenyan Somali women with female genital mutilation: a qualitative study
Source: BMC Int Health Hum Rights. 2020 Jan 28;20:3. doi: 10.1186/s12914-020-0222-6 (PMC6986153; doi:10.1186/s12914-020-0222-6)
Supplement: Supplementary file 3 — Additional file 3. In depth interview guide for Husbands, Male partners (Medicalization/shifts in FGM/C). [file 12914_2020_222_MOESM3_ESM.doc]

# **Additional File 3: In depth interview guide for Husbands, Male partners (Medicalization/shifts in FGM/C)**

In depth interview (designed to illuminate FGM/C-related complications and barriers to seeking care)

In depth interview guide for families that have experience with FGM/C. Please use this guide to facilitate the in depth interview with members of community that have experienced FGM/C. The IDI will elicit information concerning knowledge, complications and barrier to seeking services for the complications. The information collected should be backed up by the participants’ experience/evidence.

**Individual husband, male partner**

In-depth interview number:

In-depth Interview date:

Narrative Interviewer’s name:

Ethnicity of the participant:

Gender of the participant:

Age of participant:

Marital status of participant:

Number of children (boys/girls) of the participant:

Number of years lived in community:

Other relevant demographic information of participant:

Language in which narrative interview was undertaken:

Informed Consent given by participant (Purpose of the interview and confidentiality explained. Informed Consent forms signed and collected:

Consent given by participant to audio record the narrative interview:

Participants’ copy of informed consent form given:

***Introduction:***

1. Please tell me a little bit about yourself and your role/position/status in your family?

***Knowledge of FGM/C:***

*[NOTE: This section should provide information concerning the participant’s knowledge of FGM/C.]*

1. What would you say about FGM/C in your community? (Explore issues as appropriate without placing participant in a situation where they feel uncomfortable).
2. What is the principal type of FGM /C performed in your community?
3. Why do girls have to go through FGM/C
4. Where do people go to have FGM/C done?
5. What is the justification for FGM/C in your community? [**PROBE FOR**: If marriage, religion, and social pressure are mentioned, probe what beliefs underpin each of these and whether they think they are still relevant today?]
6. What complications have your wife or daughter experienced or come across or heard associated with FGM. **PROBE FOR:** Immediate complications; Gynaecological complications; Urological complications; Obstetric complications; Sexual complications; Psychological complication; Social complications
7. Where did/do you take women or girls with FGM-related complications to seek help for their problems? PROBE: Traditional healers; Public health facilities; Private health facilities; Call health personnel to the girls/woman house, Stay at home
8. What barriers did you encounter while seeking medical help for your wife/daughter from public health facilities because of FGM related complications? Probe for: cost, distance, timeliness, attitude of health care providers, others
9. What do you suggest should be done to health facilities so that more women with FGM/C complications can seek for help? PROBE FOR:
10. Do you have any comments you would like to add?
