# Supplementary material for: Exploring barriers to seeking health care among Kenyan Somali women with female genital mutilation: a qualitative study
Source: BMC Int Health Hum Rights. 2020 Jan 28;20:3. doi: 10.1186/s12914-020-0222-6 (PMC6986153; doi:10.1186/s12914-020-0222-6)
Supplement: Supplementary file 4 — Additional file 4. Key informant interview guide for community leaders (Medicalization/shifts in FGM/C). [file 12914_2020_222_MOESM4_ESM.doc]

# **Additional File 4: Key informant interview guide for community leaders (Medicalization/shifts in FGM/C)**

In depth interview (designed to illuminate FGM/C-related complications and barriers to seeking care)

Key informant interview (KII) guide for leaders that have encountered FGM/C-related cases. Please use this guide to facilitate the KII with community leaders experienced in FGM/C issues. The KII will elicit information concerning knowledge, complications and barrier to seeking services for the complications. The information collected should be backed up by the participants’ experience/evidence.

**Community leader**

In-depth interview number:

In-depth Interview date:

Narrative Interviewer’s name:

Ethnicity of the participant:

Gender of the participant:

Age of participant:

Marital status of participant:

Number of children (boys/girls) of the participant:

Number of years lived in community:

Other relevant demographic information of participant:

Language in which narrative interview was undertaken:

Informed Consent given by participant (Purpose of the interview and confidentiality explained. Informed Consent forms signed and collected:

Consent given by participant to audio record the narrative interview:

Participants’ copy of informed consent form given:

***Introduction:***

1. Please tell me a little bit about yourself and your role/position/status in the community?

***Knowledge of FGM/C:***

*[NOTE: This section should provide information concerning the participant’s knowledge of FGM/C.]*

1. What would you say about FGM/C in this community?
2. What is the principal type of FGM /C performed in the community?
3. Why do girls have to go through FGM/C
4. Who performs the FGM/C in this community?
5. What is the justification for FGM/C in your community? [**PROBE FOR**: marriageability, religion, and social pressure, probe what beliefs underpin each of these and whether they think they are still relevant today?]
6. What complications do women/girls who have undergone FGM/C experience?

**PROBE FOR:** Immediate complications; Gynaecological complications; Urological complications; Obstetric complications; Sexual complications; Psychological complication; Social complications

1. What challenges do you think women/girls with FGM/C face while seeking medical help from health facilities? **Probe for**: cost, distance, timeliness, attitude of health care providers, others
2. What do you suggest should be done to improve the health facilities functioning so that more women with FGM/C complications can seek for help? Let them discuss freely, then pick the points they raise and seek their clarification.
3. Do you have any comments you would like to add?
